# Revealing Shared and Distinct Genes Responding to JA and SA Signaling in Arabidopsis by Meta-Analysis

**DOI:** 10.3389/fpls.2020.00908

**Published:** 2020-06-26

**Authors:** Nailou Zhang, Shuang Zhou, Dongyan Yang, Zhijin Fan

**Affiliations:** State Key Laboratory of Elemento-Organic Chemistry, College of Chemistry, Nankai University, Tianjin, China

**Keywords:** plant immunity, salicylic acid, jasmonic acid, meta-analysis, co-expression network, systems biology, molecular markers

## Abstract

Plant resistance against biotrophic and necrotrophic pathogens is mediated by mutually synergistic and antagonistic effects of salicylic acid (SA) and jasmonic acid (JA) signals. However, the unique and shared genes responding to the defense mediated by JA/SA signals were largely unclear. To reveal discrete, synergistic and antagonistic JA/SA responsive genes in *Arabidopsis thaliana*, Meta-Analysis was employed with 257 publicly available *Arabidopsis thaliana* RNA-Seq gene expression profiles following treatment of mock, JA or SA analogs. JA/SA signalings were found to co-induce broad-spectrum disease-response genes, co-repress the genes related to photosynthesis, auxin, and gibberellin, and reallocate resources of growth toward defense. JA might attenuate SA induced immune response by inhibiting the expression of resistance genes and receptor-like proteins/kinases. Strikingly, co-expression network analysis revealed that JA/SA uniquely regulated genes showing highly coordinated co-expression only in their respective treatment. Using principal component analysis, and hierarchical cluster analysis, JA/SA analogs were segregated into separate entities based on the global differential expression matrix rather than the expression matrix. To accurately classify JA/SA analogs with as few genes as possible, 87 genes, including the SA receptor *NPR4*, and JA biosynthesis gene *AOC1* and JA response biomarkers *VSP1/2*, were identified by three feature selection algorithms as JA/SA markers. The results were confirmed by independent datasets and provided valuable resources for further functional analyses in JA- or SA- mediated plant defense. These methods would provide cues to build a promising approach for probing the mode of action of potential elicitors.

## Significance Statement

The potential genes, responsible for the divergence, convergence, and antagonism of JA/SA signalings in *Arabidopsis thaliana*, were systematically revealed. Insights into the JA/SA signaling cross-talk could improve the fundamental knowledge of plant immune system.

## Introduction

Plants, as sessile organisms, are constantly threatened by a diverse and variable array of pathogens and herbivores occurring simultaneously or sequentially. The phytohormones SA and JA are believed to form the hormonal backbone of plant immunity against biotic invaders (Shigenaga et al., 2017). SA is a primary defense hormone, mediating resistance against biotrophic and hemibiotrophic pathogens (feeding on living tissue). In contrast, plants primarily activate JA pathway in response to wounding caused by insects and infection by necrotrophic microbes (feeding on dead tissue) (Pieterse et al., 2012). Antagonistic and synergistic effects between JA/SA signaling pathways are considered to provide plants with a regulatory potential to survive under their complex biological environments in a resource cost-effective manner (Thaler et al., 2012; Coolen et al., 2016). This manner can be realized by shifting defense responses to either the SA- or JA- signaling pathway according to the lifestyle of the particular invading pathogen (Pieterse et al., 2012). Thus, identification of the genes related to the cross-talk of JA/SA signals in plants is of importance to develop plants with an increased disease resistance property (Shigenaga et al., 2017).

Unlike many evidence showing mutually antagonistic effects between the SA and JA signaling, their cooperation gets little attention. There is limited evidence for the shared molecular mechanisms of enhanced defense versus growth inhibition when exogenous JA/SA analogs are applied to plants (Vos et al., 2013). Although Schenk et al. have revealed that more than 50 defense-related genes are co-induced by SA and JA through microarray analysis at single time points, the total number of genes analyzed in this work was only approximately 7% of the complete *Arabidopsis thaliana* genome (Schenk et al., 2000). The activation of JA or SA mediated defense response is frequently at the cost of plant growth and reproduction (Huot et al., 2014; Karasov et al., 2017). Suppression of growth by JA or SA is well illustrated by constitutive defense mutants (e.g., *snc1*, *jazQ*), which typically have dwarf plant phenotypes due to elevated JA/SA accumulation or signaling (Zhang et al., 2003; Campos et al., 2016). The application of exogenous JA/SA analogs also arrests plant growth (Attaran et al., 2014; Huot et al., 2014). But the shared JA/SA response genes with negative affection on growth are limited. The trade-offs between JA-dependent defense against necrotrophic pathogens or insects and SA-dependent resistance against biotrophic pathogens have been well documented (Koornneef and Pieterse, 2008; Vos et al., 2013). For example, the activation of the SA pathway by biotrophic pathogen *Hyaloperonospora parasitica* strongly inhibits JA-mediated defenses against the attack of caterpillars of a small cabbage white *Pieris rapae* (*P. rapae*) (Koornneef and Pieterse, 2008). However, only a few gene expression markers, such as *WRKY70*, *WRKY62*, *GRX480*, *COI1*, *MYC2*, have been suggested to play roles in SA-JA cross-talk (Koornneef and Pieterse, 2008; Vos et al., 2013). What is more, it is difficult to infer which defense strategies plants would adopt when challenged by elicitors or pathogens just based on the transcription levels of JA/SA biomarker genes such as *PDF1.2* or *PR1* without the assistance of related mutants (Koornneef and Pieterse, 2008). The expression status of *PDF1.2* and *PR1* are dependent on the relative concentrations of SA and JA. Co-application of 10 μmol/L JA and 250 μmol/L SA has a synergistic effect on the transcription of *PDF1.2*. Co-application of 10 μmol/L SA with 500 μmol/L enhances the accumulation of *PR1* transcripts. Co-application of 50 μmol/L SA with 50 μmol/L JA at 12 h after treatment has a maximally synergistic effect on the transcription of *PDF1.2* and *PR1* (Mur et al., 2006).

The availability of growing genome-wide transcriptome datasets treated by JA/SA and their functional analogs provides an unprecedented opportunity to probe the intricate nature of JA/SA cross-talk defense against biotrophic and necrotrophic pathogens. Meta-Analysis provides a powerful strategy to exploit original findings with higher statistical power by combining similar scientific studies. For instance, by performing Meta-Analysis on rice microarray studies under abiotic and biotic conditions, Shaik and Ramakrishna (2014) identified candidate genes for broad-spectrum resistance in rice. Machine learning algorithms like principal component analysis (PCA), hierarchical clustering analysis (HCA), and support vector machines (SVM), provide effective methods for classification of two or more data categories. Furthermore, feature selection procedures, such as recursive feature elimination SVM (RFE-SVM), recursive-SVM (R-SVM) and random forest (RF) provide ways to identify the best biomarkers to discriminate data categories with higher accuracy (Johannes et al., 2010, 2011; Shaik and Ramakrishna, 2014).

In this study, JA/SA analogs were classified into two categories by PCA and HCA based on the global differential gene expression matrix. The shared and unique JA/SA signaling genes (i.e., uniquely up/down-regulated, or oppositely/same directional regulated by two signals) were identified by performing Meta-Analysis on the expression profiles of JA/SA analogs. The best gene expression markers, which could distinguish JA/SA analogs with higher sensitivity, were identified according to RF, R-SVM, and RFE-SVM. By comparing the gene expression patterns during biotrophic and necrotrophic pathogens to those induced by JA/SA analogs, the candidate genes related to broad-spectrum resistance against pathogens were identified. The robustness of these methods was confirmed by reproducing the results originating from the RNA-Seq data through using independent microarray data with the same data processes.

## Materials and Methods

### Data Collection and Procession

The RNA-Seq transcriptome datasets through treatments of JA/SA analogs or pathogen infections were publicly available from NCBI SRA database and adopted in this study. The datasets in the unpublished articles or without clearly annotated treatment or infection time were removed. As results, the raw data files (sra) of 13 studies [PRJNA224133 (SA, MeJA, mock), SRP041507 (COR, mock), PRJNA270886 (COR, mock), PRJNA354369 (BTH, MeJA, mock), PRJNA303108 (DPMP, mock), PRJNA394842 (INA, mock), PRJNA318266 (MeJA, mock), PRJNA348676 (wild type + *Pto* DC3000, *deps* + *Pto* DC3000, mock), PRJNA354373 (*Pto* DC3000, mock), PRJNA276445 (*B. cinerea*, mock), PRJNA315516 (*B. cinerea*, *P. rapae*, mock), PRJNA418121 (*S. sclerotiorum*, mock), PRJNA336058 (TCV, mock)] with 416 samples were downloaded for this work. Raw reads were aligned to TAIR10 genome release using Hisat2 v.2.0.4 (Kim et al., 2015) with default parameters. Gene expression levels were calculated using FeatureCounts (Liao et al., 2014) and were normalized by the voom normalization method in limma v.3.32.8 (Ritchie et al., 2015). Differential gene expressions were assessed by calculating the difference in absolute expression between matched treatment and mock samples. The samples of the early time (1, 2, 3, 4, 6 h) after infection with *Pto* DC3000 were excluded because these samples had almost no significant differential expression genes as reported (Mine et al., 2018).

The DNA microarrays used belong to the Affymetrix platform GPL198. The selection criteria for datasets were the same as described above. As results, their CEL files of 12 studies [GSE39384 (MeJA, mock), GSE10732 (OPDA, mock), GSE51626 (SA, mock), GSE22942 (SA, mock), GSE10646 (BTH, mock), GSE13833 (DCA, INA, mock), E-MEXP-3122 (*S. sclerotiorum*, mock), GSE16497 (aphid, mock), GSE5684 (*B. cinerea*, mock), GSE50526 (*A. brassicicola*, mock), GSE17500 (*Pto DC3000*, mock), GSE5520 (*Pto DC3000*, mock)] with 112 samples were downloaded from GEO or EBI and normalized by RMA method. Based on the annotation file for GPL198, the ID of probesets was converted into TAIR gene locus ID. Differential gene expression was performed with limma (Ritchie et al., 2015).

### Wigwams Module Mining

Wigwams program (Polanski et al., 2014) was used to identify modules of co-expressed genes spanning mock, SA and JA time course datasets of PRJNA224133. Wigwams program was run with default parameters (Set Sizes: 50:50:250; Alpha:0.05; Correlation Net: 0.7; Merging-Overlap proportion: 0.3; Merging-Mean Correlation: 0.9; Merging-Correlation Filter: 0.8; Sweeping-Overlap proportion: 0.5 with run Merged modules; Remove small modules with size threshold: 10;10;8;5;5).

### Meta-Analysis

In order to perform Meta-Analysis, each dataset was preprocessed as described above and normalized. The Meta-Analysis was implemented by the MetaDE R package (Ma et al., 2019) based on SA-mock and JA-mock publicly accessible RNA-Seq datasets to identify the robust up/downregulated genes, respectively. Fisher exact test was used and a *p*-value of < 0.05 was set as the cutoff. Then, overlapping genes in the four lists (SA up/down and JA up/down) were identified as discrete, synergistic and antagonistic JA/SA responsive genes in *Arabidopsis thaliana*.

### Gene Ontology (GO) Analysis

To explore the biological interpretation of the genes identified in Meta-Analysis and co-expression modules, we did GO analysis with g:Profiler (Raudvere et al., 2019), of which the database synced by new data from Ensembl.

### The Removal of Batch Effects

The removal of batch effects was implemented by the ComBat method of sva v.3.26 based on normalized gene expression matrix of RNA-Seq JA/SA signals samples from different projects.

### HCA and PCA

HCA and PCA were implemented to visualize the structures of datasets. All HCAs were conducted with the complete linkage hierarchical clustering method and Euclidean distances, and were visualized as trees. PCAs were performed using the base R function prcomp.

### Feature Selection Methods

To identify as fewer genes as possible to robustly distinguish JA/SA signals, three published gene selection methods were employed on differential expression matrix of antagonistic JA/SA responsive genes. SVM-RFE was an iterative SVM-based wrapper algorithm, which systematically assessed the prediction performance of feature subsets using the implementation in the R-package pathClass (Johannes et al., 2011). The implementation of pathClass was directly based on the minimum eigenvalue of 20% of the Laplace matrix of the normalized graph and the corresponding eigenvectors were retained to calculate the kernel matrix (Johannes et al., 2011). Here, SVM-RFE parameters were cross-verified internally (10-folds) by nested cross-validation, and the generalization error was estimated by the external loop (100 repeats of 10-folds) with regularization parameter C of values ranging from 10^–3^ to 10^3^. R-SVM was another recursive SVM classification method using different feature subsets and selected the best-performing features according to the cross-validation error rates (Zhang et al., 2006). R-SVM algorithm was similar to SVM-RFE but selected important features based on frequency. To determine the accuracy of the classification, R-SVM classification was performed by utilizing a leave-one-out cross-validation program, randomly dividing the features into the training set and test set, and recursively eliminating the features with poor performance and high cross-validation error rate. RF was an algorithm based on the decision tree, which grew branches of classification tree set by randomly selecting feature subset from guiding sample, and carried out class prediction based on majority voting of the set. RF measured the importance of variables based on the degree of accuracy degradation when variables were excluded. In RF, it was not necessary to split the data set split into test data and training data to test the accuracy. RF conducted an internal validation as two-thirds of the available training data was used to grow each tree and the remaining one-third of training data was always used to calculate out-of bag error to evaluate model performance. The randomForest package in R was implemented to identify the most 15 important variables as biomarkers.

### Statistical Analyses

All statistical analyses were implemented in R v.3.4.2 (R Core Team, 2015).

## Results

### JA/SA Analogs Were Discriminated as Two Classes Based on Differential Gene Expression Profiles

**FIGURE 1:**
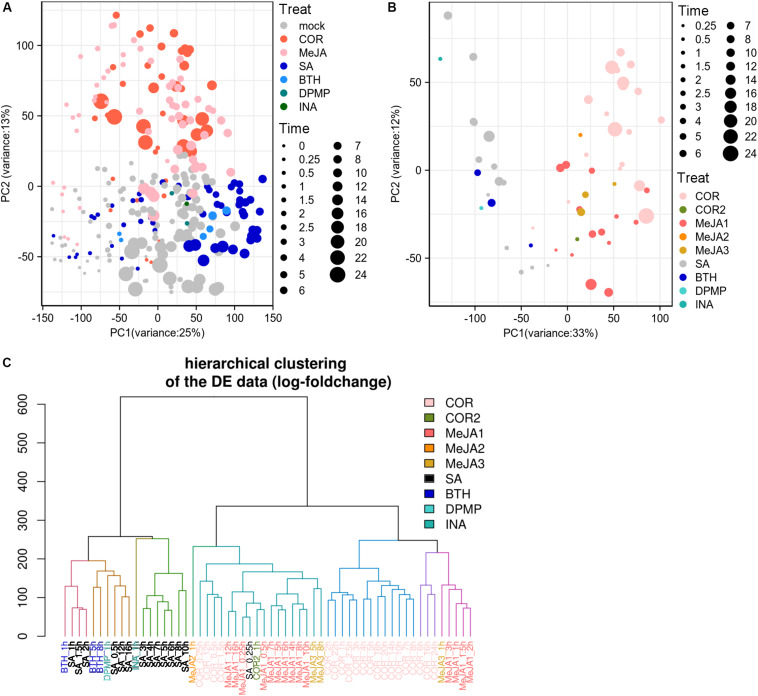
JA/SA analogs were discriminated as two classes based on global differential gene expression matrix. **(A,B)** PCA plots showed the first 2 principal component diagram based on global gene expression matrix (after removing bath effects) and differential gene expression matrix. **(C)** HCA dendrogram profiled classification of global gene differential expression. Symbol sizes increased with time (hours) after the application of JA/SA analogs. The MeJA1-3 and COR/2 represented datasets from different laboratories. The RNA-Seq datasets: SRP041507 (COR); PRJNA224133 (SA/MeJA1); PRJNA354369 (BTH/MeJA2); PRJNA318266 (MeJA3); PRJNA270886 (COR2); PRJNA303108 (DPMP); PRJNA394842 (INA).

The previous researches showed that the a/biotic stresses and tissue types were the primary factors driving corresponding datasets into separate entities based on gene expression landscape (Shaik and Ramakrishna, 2014; He et al., 2016). We wondered if the JA/SA functional analogs could be grouped into two classes based on the global gene expression matrix since both phytohormones were traditionally thought to be mutually antagonistic. PCA was implemented to explore the data structures using publicly available RNA-Seq gene expression profiles generated from plants treated with JA analogs [coronatine (COR), MeJA], SA analogs (benzo-(1,2,3)-thiadiazole-7-carbothioic acid S-methyl ester (BTH), 2,4-dichloro-6-{(E)-[(3-methoxyphenyl) imino]methyl}phenol (DPMP), 2,6-dichloro-isonicotinic acid (INA), SA), and mock (Supplementary Table S1). However, to our disappointment, the batch effects from separate laboratories were the biggest driving factor to segregate the datasets (Supplementary Figure S1). The post-stimulus time seemed to be the second factor (Supplementary Figure S1B). Moreover, batch effects completely compromised the biological interpretation of the data. After removing the batch effect, the samples of JA analogs were separated from those of SA analogs and mock (Figure 1A). But there was a lot of overlap between SA analogs and mock (Figure 1A). Then PCA and HCA were performed to explore the data structures of the global differential expression matrix. The samples of JA/SA analogs were classified into two separate subgroups in PCA and HCA (Figures 1B,C). The synthetic plant defense elicitors BTH and INA were traditionally thought to mimic the defense-related effect of SA (Bektas et al., 2016), and the newly identified synthetic defense elicitor DPMP, which was a partial agonist of SA (Bektas et al., 2016), was also clustered with SA (Figures 1B,C). Coronatine (COR), a structural mimic of JA-Ile (Attaran et al., 2014), was clustered with JA. These results indicated that it might be a promising method to predict the mode of action of potential plant elicitors by unsupervised machine-learning methods like PCA and HCA based on global differential expression matrix.

### Shared and Unique Biological Processes Responsible for JA/SA Signals Identified by Meta-Analysis

**FIGURE 2:**
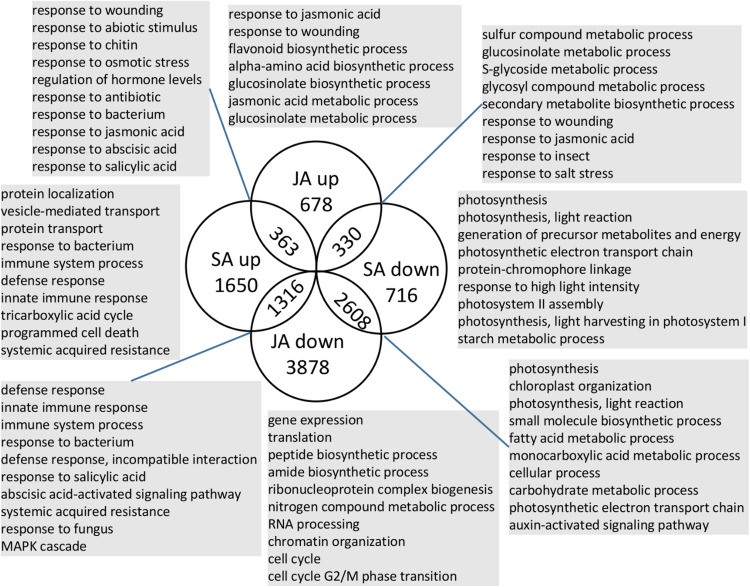
Number and function of shared and unique genes regulated by JA/SA signaling. Selected enriched GO terms are shown for each subset of genes. Full data set for JA/SA regulated genes were presented in Supplementary Table S5.

To investigate which biological processes and genes were the unique or shared response to JA/SA signals, Meta-Analysis was applied to the publicly available RNA-Seq experiments that had at least two biological replicates of the mock and JA/SA treatment samples, of which 120 samples (two sub-series from PRJNA224133; two sub-series from PRJNA224133; and PRJNA303108) were used for SA-mock Meta-Analysis and 195 samples (two sub-series from PRJNA224133; two sub-series from PRJNA224133; SRP041507; and PRJNA270886) were used for JA-mock Meta-Analysis (detail in Supplementary Table S1). As a result, there were 9173 and 6983 significant differential expression genes, respectively, responding to JA and SA analogs identified by Fisher’s method from R package MetaDE (Ma et al., 2019) with the adjusted *p*-value. Overlapping genes in the four lists (SA up/down and JA up/down) were identified (Figure 2 and Supplementary Table S2). The majority (approximately 60%) of these genes was uniquely regulated by JA or SA signaling; 26% of these genes were regulated by JA/SA in the opposite direction; at least 14% of these genes were regulated by JA/SA in the same direction.

**FIGURE 3:**
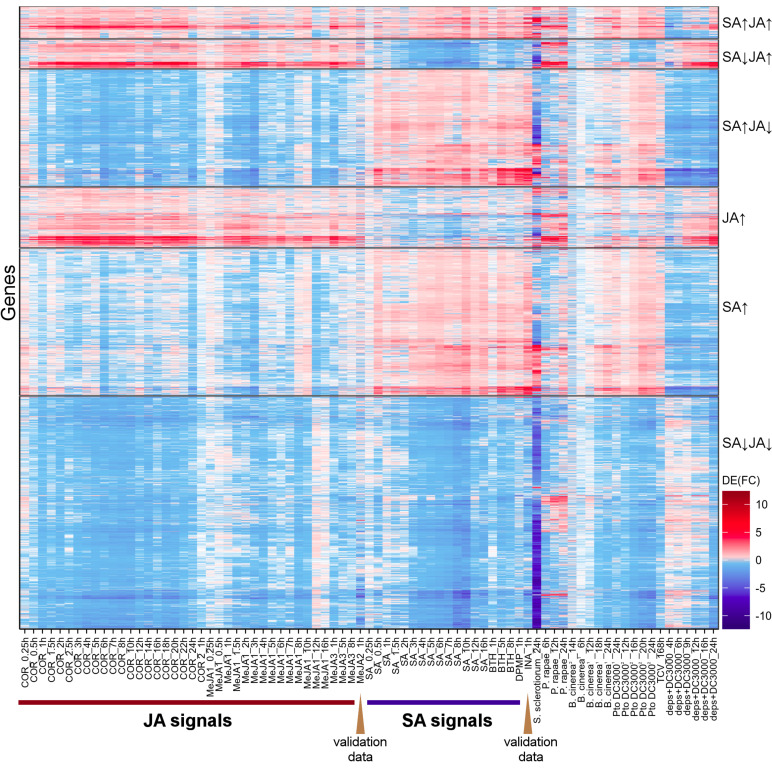
Differential gene expression dynamics of plants challenged by JA/SA analogs and various pests. Heatmap depicted the differential gene expression profiles, challenged by JA/SA analogs, biotrophic and necrotrophic pathogens. *S. sclerotiorum* (PRJNA418121); *P. rapae* (PRJNA315516); *B. cinerea*^1^ (PRJNA315516); *B. cinerea*^2^ (PRJNA276445); *Pto* DC3000^1^ (PRJNA354373); *Pto* DC3000^2^ (PRJNA348676); TCV (PRJNA336058); *deps Pto* DC3000 (PRJNA348676). The JA/SA analogs datasets are same to Figure 1.

To test the robustness of Meta-Analysis results, the differential expression profiles of INA and MeJA RNA-Seq datasets, which were not used in Meta-Analysis and had only one biological replicate (Supplementary Table S1), and those of JA/SA analogs’ microarray datasets were exhibited in heatmap (Figure 3 and Supplementary Figure S2, and Supplementary Tables S1, S3). The plot showed that the overall differential expression pattern of RNA-Seq (INA and MeJA) and microarray datasets were consistent with the results of Meta-Analysis (Figure 3 and Supplementary Figure S2). This suggested the reliability and robustness of the Meta-Analysis results.

363 genes, co-induced by SA and JA signals (SA↑JA↑; Figure 2), were enriched in ontologies involving response to wounding, JA, nematode, SA, fungus/bacterium, abscisic acid, and abiotic stimulus, and JA biosynthetic process (Figure 2 and Supplementary Table S4). A detailed examination of these genes revealed several known defense regulators: *WRKY18*/*40*, *EDS5*, *ZAT10/12*, *ERF1A*, *AtrbohF*, *PEN3*. *WRKY18/40* positively modulated the expression of JA-signaling genes by partly suppressing the expression of JAZ repressors (Pandey et al., 2010). *EDS5* was necessary for SA accumulation after a/biotic stress and functioned as a multidrug and toxin extrusion-like transporter in the export of SA from the chloroplast to the cytoplasm in *Arabidopsis thaliana* (Serrano et al., 2013). *ZAT10/12* both belonged to C1-2i subclass family C2H2 zinc finger TFs in *Arabidopsis thaliana* and were highly induced by cold, osmotic, salt, drought, UV-B, and wounding in shoot tissue (Yin et al., 2017). *ERF1* expression was transcriptionally controlled by *EIN3* and might be a regulator of *PDF1.2* (Solano et al., 1998). *AtrbohF* was required for accumulation of reactive oxygen species during plant defense (Torres et al., 2002). *PEN3* is an essential component of cell wall defense linked to callose deposition and transportation of glucosinolate (Clay et al., 2009). Thus, these genes might play shared roles for JA/SA pathway to regulate plant defense. It is well known that the establishment of plant defenses is often accompanied by the suppression of growth (Huot et al., 2014; Karasov et al., 2017). Additional inspection revealed activation of two growth inhibitors: *RAV1*, *ZAT6* (Hu et al., 2004; Devaiah et al., 2007), and two well documented molecular switches (*ATAF2*, *ERF2*) for plant growth-to-defense transition (McGrath et al., 2005; Peng et al., 2015, p. 2), suggesting underlying shared mechanisms by which JA/SA regulate growth-defense tradeoffs. Another hypothesis emerging from the analysis of JA/SA co-induced genes involved JA biosynthetic genes (*LOX3*, *AOC3*, *OPCL1*, and *ACX1*). The synthesis of JA began with the esterified α-linolenic acid on chloroplasts. Then, 12-oxo-phytodienoic acid (OPDA) was synthesized in the chloroplast from α-linolenic acid through a series of consecutive reactions catalyzed by *LOX*, *AOS*, and *AOC.* Subsequently, OPDA was converted to JA-CoA, a precursor of JA, catalyzed by *OPR3, OPCL1*, and *ACX* (Kaur et al., 2009). Thus, SA might have a positive effect on the accumulation of JA.

Our results revealed that 71% (2608 genes; Figure 2) of SA down-regulated genes were also repressed by JA (SA↓JA↓). The ontologies representing photosynthesis-related component, carbohydrate metabolic process, plant organ development, and auxin transport, metabolic, and signaling process were enriched in this group (Figure 2 and Supplementary Table S4). The most striking gene class co-repressed by JA/SA signals included 98 photosynthesis genes (corrected *P*-value < 6.1 e^–25^), which accounted for more than one-third of all photosynthetic genes (Figure 2 and Supplementary Table S4). Among these 98 genes, 11 genes (e.g., *RBCS1B*/*2B*/*3B*) were involved in the Calvin cycle (Supplementary Table S4). *RBCS1B/2B*/*3B* coded ribulose-1,5−bisphosphate carboxylase/oxygenase (RuBisCO). RuBisCO constituted 30–50% of the total soluble protein in C3 plants and functioned as a potential N storage protein. Nitrogen investment in RuBisCO and total soluble proteins were reduced by 89% in young rosette leaves of *Nicotiana attenuata* after herbivore attacks (Havko et al., 2016). Here, RuBPCase activase (RCA), which modulated the activity of RuBPCase, was co-repressed by JA/SA analogs. RCA-silenced plants reduced photosynthetic and growth (Mitra and Baldwin, 2014). A detailed inspection of these group genes revealed four genes (*BHLH93*, *ATH1*, *DAG2*, and *HB25*) related to gibberellin biosynthetic and 42 genes involved in the auxin-activated signaling pathway. These indicated that SA and JA signaling had the shared negative effects on growth, such as diminishing photosynthesis, inhibiting the auxin and gibberellin pathway, and reallocating resources of growth toward defense.

Around 40% (1316 genes) of the SA up-regulated genes were repressed by JA (SA↑JA↓; Figure 2). The ontologies representing innate immune response, programmed cell death, plant-type hypersensitive response, response to salicylic acid, and MAPK cascade were enriched in this group (Figure 2 and Supplementary Table S4). The most striking gene class in this group included 64 disease resistance genes with a nucleotide binding site (NBS), which play vital roles in resistance against pathogens. *Arabidopsis thaliana* has 167 NBS-encoding R genes (Yu et al., 2014). Here, 32 TIR R proteins (TIR-NBS-LRR) and 6 coiled coil type R proteins (CC-NBS-LRR) were induced by SA and inhibited by JA (Supplementary Table S2). R genes, which specifically recognized with corresponding pathogen a/virulence genes, provided partial and sometimes even full resistance, and activate effector-triggered immunity (ETI). Two other notable features in this group were 25 out of 223 of leucine-rich repeat receptor-like kinases (LRR-RLKs), and 10 out of 57 receptor-like proteins (At-RLPs) (Supplementary Table S2). RLPs/RLKs in the apoplast could perceive pathogen-associated-molecular-patterns (PAMPs) and effectors directly or indirectly, leading the activation of PAMP-triggered immunity (PTI) (Wang et al., 2008; Gou et al., 2010). Additionally, *PAD4*, *EDS16/ICS1*, *CBP60G*, *SARD1*, which were vital for SA accumulation (Zhang and Li, 2019), were induced by SA and suppressed by JA. It was well documented that the SA biosynthesis gene *ICS1* was inhibited by NAC TFs mediated by JA (Zheng et al., 2012). Therefore, JA might attenuate SA induced immune response by inhibiting the accumulation of SA, the expression of R and RLPs/RLKs genes.

Approximate 24% (330 genes) of the JA up-regulated genes were repressed by SA (SA↓JA↑; Figure 2). This group overrepresented for functional terms associated with JA defense responses, S-glycoside biosynthetic process, and biosynthesis of secondary metabolites (Figure 2 and Supplementary Table S4). Notably, this group included three essential JA biosynthesis genes (*DDE2*, *AOC1/4*) and two JA response biomarker genes (*VSP1/2*). This observation was consistent with that JA biosynthesis gene *DDE2* was suppressed by SA (Leon-Reyes et al., 2010). 11 out of 22 glucosinolate biosynthesis pathway genes were induced by JA and repressed by SA. Glucosinolates were secondary metabolites derived from a variety of amino acids, and were biologically inactive in their intact form. Upon wounding, glucosinolates could be hydrolyzed to an array of biologically active compounds such as isothiocyanates, which were deterrent to most herbivores (Falk et al., 2014). Previous efforts showed that SA did not affect JA biosynthesis, but it affected the downstream genes of COI1-dependent JA signals at the level of transcriptional regulation (Leon-Reyes et al., 2010; der Does et al., 2013). Therefore, the biosynthesis pathway of secondary metabolites, which was regulated by JA, might be the target for SA-mediated antagonism.

Nearly half of the SA upregulated genes were uniquely activated by SA (SA↑; Figure 2). These group genes (1650 genes) were enriched for the annotation term “response to bacterium,” “immune system process,” “programmed cell death,” “response to cadmium ion,” and “response to oxidative stress” (Figure 2 and Supplementary Table S4). The most striking ontologies enriched in this group were “localization (334 genes),” “transport (324 genes),” “protein transport (162 genes),” “protein metabolic process (397 genes),” “phosphorus metabolic process (214 genes),” and “response to metal ion (64 genes)” (Supplementary Table S4). However, these genes appeared to less associated with SA mediated defense inferring from GO biological processes enrichment analysis by g:Profiler. On the other hand, the remaining 854 genes in this group were closely related to defense mediated by SA and included 12 WRKY transcription factors (TFs), 11 bZIP TFs, 7 NAC genes, 6 R genes, 4 RLP genes, and SA response genes *TGA3* and *PR1* (Supplementary Table S2). Indeed, over 40 out of 72 *Arabidopsis thaliana* WRKY TFs were responsive to SA treatment (Dong et al., 2003). Interestingly, although these WRKY TFs were uniquely induced by SA, their roles in JA/SA cross-talk might be complex. For example, *WRKY70* acted as an activator of SA-dependent defense genes while repressing the JA response (Koornneef and Pieterse, 2008); The overexpression of *WRKY33* resulted in decreased *PR1* expression and enhanced susceptibility to *Pto* DC3000, and *wrky33* mutant plants exhibited reduced *PDF1.2* expression and increased susceptibility to the necrotrophs *Botrytis cinerea* (Koornneef and Pieterse, 2008).

Nearly half of the JA upregulated genes were uniquely regulated by JA (JA↑; Figure 2). Broad annotations such as “response to wounding,” “response to JA,” “secondary metabolic process,” “glucosinolate metabolic process,” “flavonoid metabolic process,” and “negative regulation of nucleic acid-templated transcription” were presented in this group (Figure 2 and Supplementary Table S4). The genes in this group included many known genes involved in JA pathway, for example, *AOC2*, *OPR3*, *LOX1*, *LOX2*, *JMT*, *JAZ2/3/5/6/7/10/12*, *MYC3*, *PDF1.2*, and the DELLA protein *RGL3* (Supplementary Table S2). Interestingly, two genes (*EPS1*, *MYB44*) were found induced by JA, but have negative effect on defense against necrotrophic fungi and have positive effect on biotrophic pathogen. *EPS1* appeared to act upstream of SA but was induced by MeJA, rather than SA. The *eps1-1* mutants showed enhanced resistance to necrotrophic fungi but displayed hyper-susceptibility to avirulent and virulent *Pto* DC3000 (Dempsey et al., 2011). *MYB44* over-expression enhanced resistance to the biotrophic pathogen by upregulating *WRKY70* and PR genes without a change of SA content (Shim et al., 2013). These suggested that some JA response genes could enhance SA-mediated immune.

Less than 20% (716 genes; Figure 2) of the SA down-regulated genes were uniquely regulated by SA (SA↓). The ontologies representing photosynthesis, generation of precursor metabolites and energy, starch metabolic process, alpha-amino acid metabolic process were enriched in this group (Figure 2 and Supplementary Table S4). Notably, 19 out of 50 proteins involved in photosystem II, 14 out of 40 proteins involved in photosystem I, 13 out of 57 photosynthetic electron transport chain were uniquely inhibited by SA. These suggested that SA might have a unique inhibitory effect on photosynthesis.

Fifty percent (3878 genes; Figure 2) of the JA downregulated genes were uniquely regulated by JA (JA↓). It was known that JA could not only regulate plant defense against necrotrophic pathogens and chewing insects, but also slowed down the progression of cell cycle and increased the number of G0/G1 phase cells by impairing G1/S transition, thus reducing the number of actively dividing cells (Huot et al., 2014). This group enriched in ontologies capturing biological processes including nitrogen compound metabolic process (1892 genes), gene expression (1083 genes), translation (346 genes), ribonucleoprotein complex biogenesis (243 genes), RNA processing (172 genes), and cell cycle (175 genes) (Figure 2 and Supplementary Table S4). Additionally, there were 21 R genes, 28 RLK genes, and 7 RLP genes presented in this group (Supplementary Table S2).

### The Context-Specific and Shared Co-expression Patterns Under JA/SA Regulation

**FIGURE 4:**
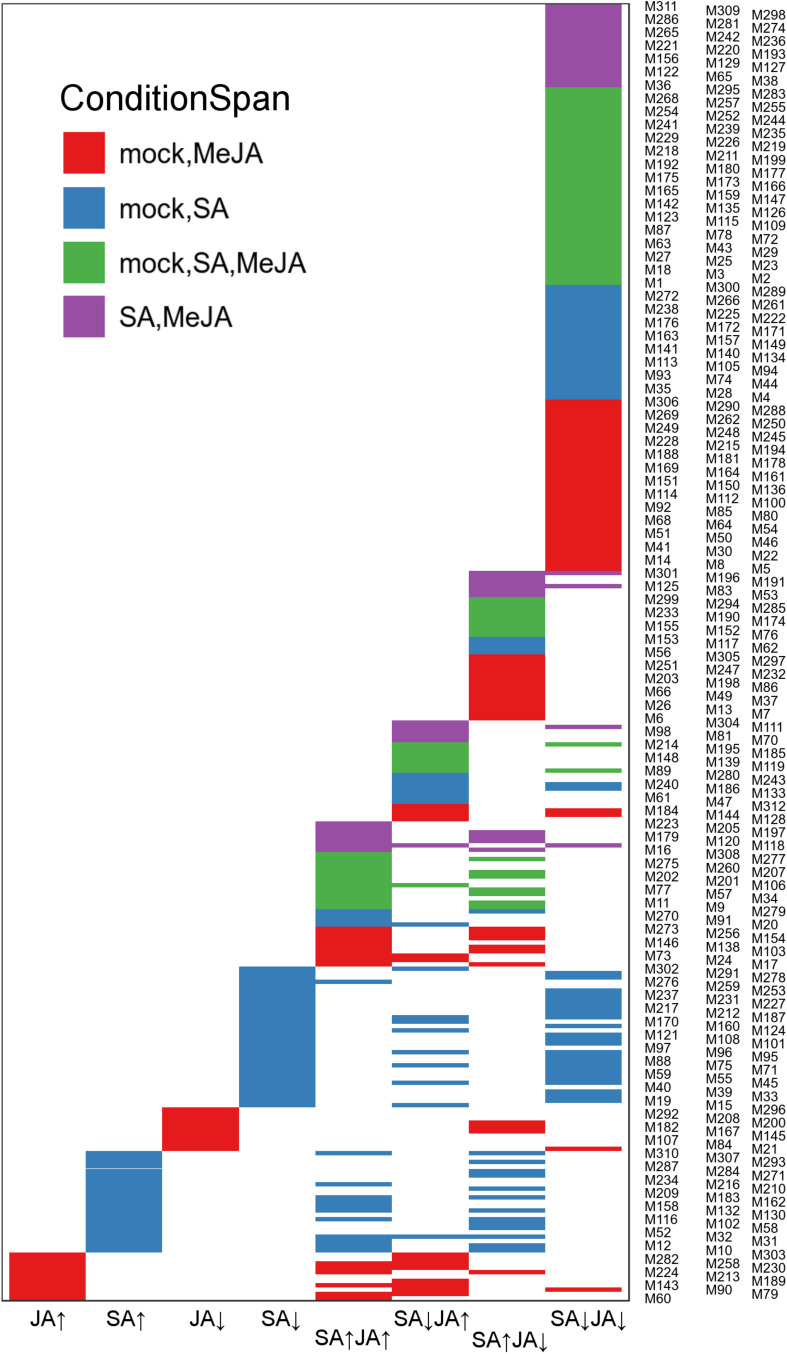
The JA/SA regulated genes of the Meta-Analysis were compared with Wigwams genes using Fisher exact test. Venn diagram depicted the overlap between JA/SA regulated genes and Wigwams genes (Fisher’s exact test, *p* < 0.05). To avoid overlapping the *y*-axis labels, the Wigwam modules were shown in three columns.

**FIGURE 5:**
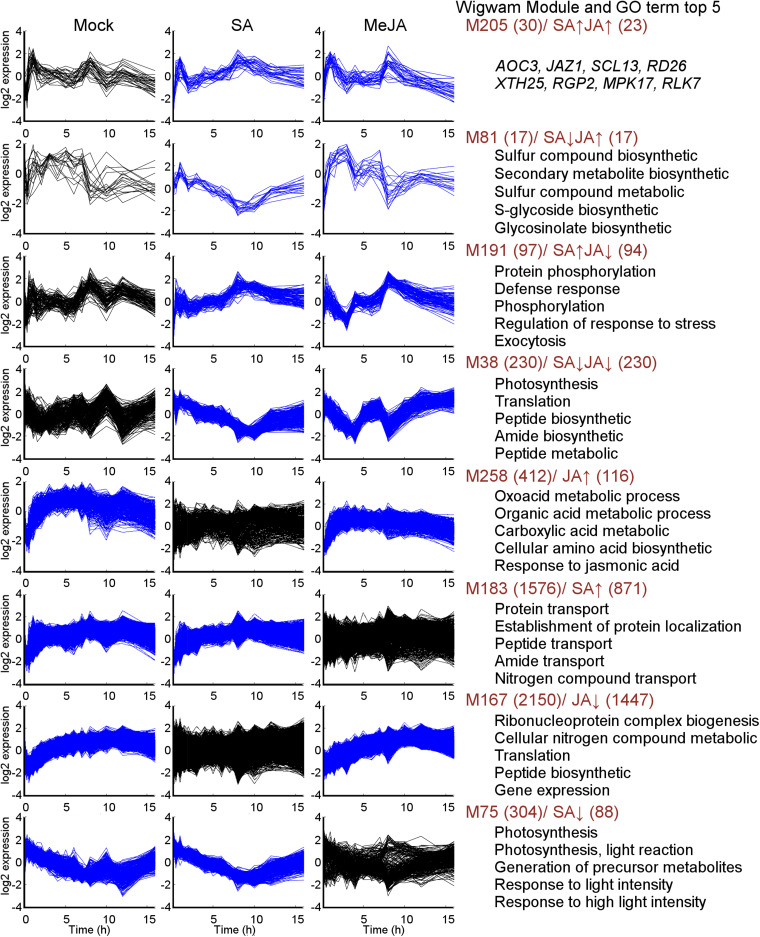
Expression patterns of selected Wigwams modules found by mining Mock, SA, and MeJA time courses. Blue–colored graphs indicated modules of which the genes showed highly coordinated co-expression over time in the given condition. In the black-colored graphs, the genes in the module were not significantly co-expressed. The number of genes of each module and JA/SA regulated genes in each module, was colored in brown. The top 5 GO terms with highest significance in the respective modules were given (full data set for selected Wigwams modules were presented in Supplementary Table S5). The *y*-axis indicates the mean normalized log2 expression levels. The *x*-axis indicates time (h) after treatment.

Generally, genes with highly coordinated expression across various situations were usually closely related in function. Co-expression networks could integrate genes with unique functions into modules with high coordination in an intuitive way. In order to identify in greater detail the gene expression pattern in response to mock, SA or JA, the Wigwams algorithm, which identified context-specific and shared co-expression patterns in two or more time-series datasets (Polanski et al., 2014), was performed based on datasets of PRJNA224133, containing three high temporal resolution datasets (SA, JA, and mock treatment). In total, 312 modules (containing 9255 unique genes; Supplementary Table S5) were identified and showed highly co-expression across at least two-time series datasets. Then, fisher’s exact test was employed to estimate overrepresentation of JA/SA regulated genes in Meta-Analysis within these Wigwams modules (Figure 4 and Supplementary Table S6). The genes in these modules were co-regulated by JA and SA in the same or opposite direction (Figure 4). For example, the JA/SA co-induced genes like *AOC3*, *JAZ1*, *SCL13* were in Wigwam module M205 (Figure 5); The genes in M81 enriched GO term associated with secondary metabolite biosynthetic process and glycosinolate biosynthetic process, and were induced by MeJA and repressed by SA (Figure 5 and Supplementary Table S4). These confirmed the hypothesis that SA suppresses JA signaling through inhibiting the downstream of JA pathway. The genes in M191 were induced by SA and repressed by JA, and contained *EDS1*, *SAG101*, 6 R genes, 6 RLK genes, and 1 RLP gene (Figure 5 and Supplementary Table S4). *EDS1* and *SAG101* regulated defense signaling mediated by R proteins (Venugopal et al., 2009). Again, these suggested that JA might attenuate the SA induced immune response by inhibiting the expression of R and RLPs/RLKs genes. The genes in M38 were co-repressed by JA and SA (Figure 5 and Supplementary Table S4). Among the 230 genes in M38, six genes coded PsbP domain proteins, which are required for photosystem II to be fully operational in *vivo* (Kochhar, 1996), and four enzymes functioned in the calvin cycle. Consistently, calvin cycle and photosynthesis were also uniformly repressed following a biotic assault (Bilgin et al., 2010). The genes in M38 had differential co-expression patterns across the time course following SA and MeJA treatment (Figure 5). These suggested that JA/SA might repress the transcription of photosynthesis genes through different mechanisms.

It was remarkable that almost all genes uniquely regulated by SA or JA showed tight co-expression across mock-SA or mock-MeJA treatment conditions, respectively (Figure 4 and Supplementary Table S4). For example, the genes in M258 showed a pattern of gradual upregulation and highly coordinated co-expression in both mock and JA treatment but maintained a stable expression pattern and lowly coordinated co-expression in SA treatment (Figure 5). 116 genes uniquely upregulated by JA were included in M258, and enriched in ontologies related to response to JA (Figure 5 and Supplementary Table S4). The presence of JA biosynthesis genes *AOC2* and *LOX2* in M258 suggested that SA did not affect JA biosynthesis. In M183, the genes uniquely upregulated by SA enriched function terms less related to SA-mediated defense, such as, “localization,” “transport,” “protein transport,” “protein metabolic process,” “phosphorus metabolic process,” and “response to metal ion” (Figure 5 and Supplementary Table S4). This was consistent with the above analysis. More than one-third JA uniquely downregulated genes were in M167, which showed tight co-expression across mock and JA treatment (Figure 5), and overrepresented for functional terms associated with gene expression, translation, ribosome biogenesis, RNA processing. Around one-fourth genes in M75, which showed tight co-expression across mock and SA treatment and repressed by SA (Figure 5), and enriched functional terms related to photosynthesis. This suggested once again that SA had a unique inhibitory effect on photosynthesis.

### The Identification of JA/SA Response Biomarkers Based on Feature Selection

**FIGURE 6:**
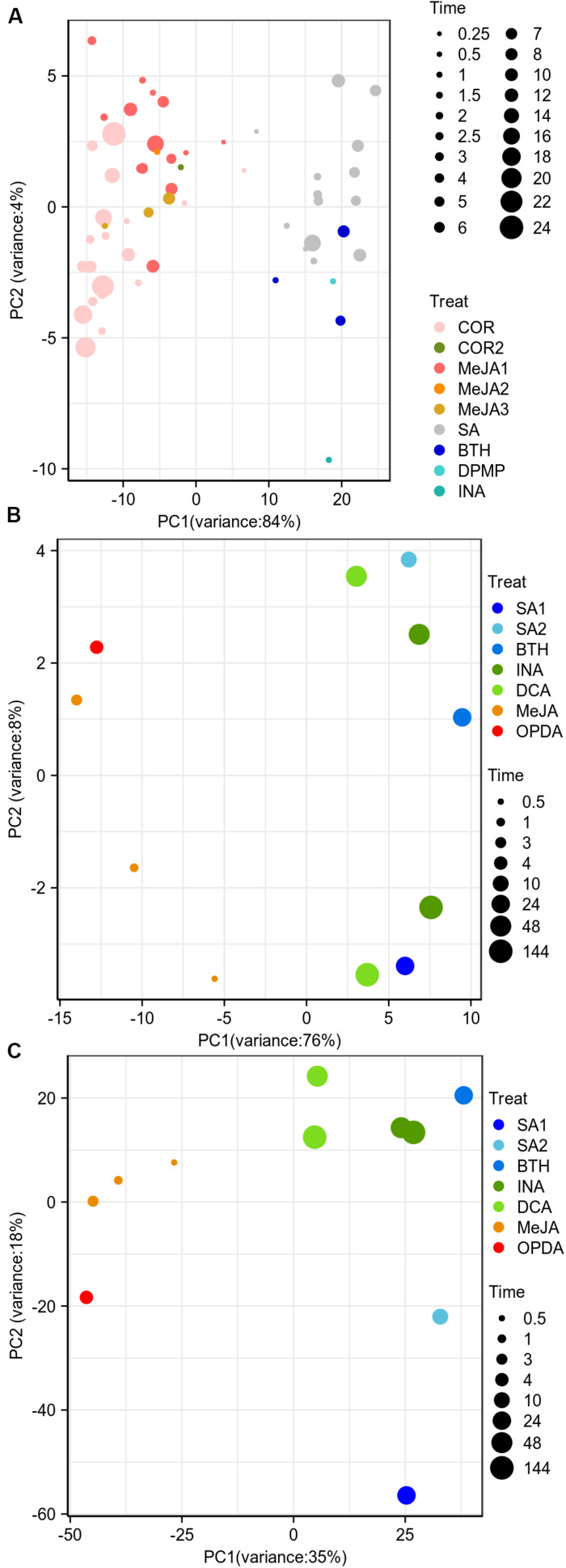
The classification effect of identified SA and JA biomarker genes. **(A,B)** The performance of PCA on the identified biomarker genes’ differential expression matrix (**A**, RNA-Seq, **B**, microarray). **(C)** PCA plots showed the first 2 principal component diagram based on differential gene expression matrix (microarray). SA1/2 represented datasets from different laboratories. SA1 (GSE22942); SA2 (GSE51626); BTH (GSE10646); INA (GSE13833); DCA (GSE13833); MeJA (GSE39384); OPDA (GSE10732). The RNA-Seq datasets are same to Figure 1.

Since most of the screening strategies of plant elicitors were based on SA-induced immune responses, this led to the fact that most of the known synthesized elicitors are functional analogs of SA, such as BTH, INA, and 3,5-dichloroanthranilic acid (DCA) (Bektas and Eulgem, 2015). Therefore, it was necessary to identify new and robust JA/SA response expression markers to screen for novel plant elicitors. In order to identify the biomarkers that distinguish JA/SA analogs with high accuracy, three feature selection algorithms: RF, R-SVM, and RFE-SVM were performed on the differential expression matrix of 1645 antagonistic JA/SA responsive genes (Supplementary Table S2). In order to improve the accuracy of classification, samples of 0.25 and 0.5 h were removed. Finally, 87 genes, which could classify JA/SA analogs with 100% accuracy, were identified (Supplementary Table S7). To test whether these identified genes could better distinguish JA/SA analogs, PCA and HCA analysis were performed based on differential expression matrix of these 87 genes (Figure 6A). The first principal component captured 84% variance between JA/SA analogs, and JA/SA analogs were grouped into the two main branches in HCA dendrogram (Supplementary Figure S3A). Furthermore, after removing unmatched genes of microarray data, 80 out of these biomarker genes were able to capture 76% variance in the first principal component to distinguish JA/SA analogs, compared with 35% for all genes (Figures 6B,C). Interestingly, the datasets of OPDA, and DCA were not used for identifying biomarkers and Meta-Analysis. OPDA, a cyclopentenone precursor of JA, functioned independently of JA/MeJA signaling pathway (Taki et al., 2005). DCA, a plant defense elicitor, triggered immune responses that were largely independent of *NPR1* and did not require the accumulation of SA (Faize and Faize, 2018). Here, OPDA and DCA were clustered with other JA/SA functional analogs, respectively (Supplementary Figure S3B).

Among these 87 genes, one SA receptor *NPR4*, two TIR-NBS-LRR R genes (*RPP4*, *AT5G41750*), two RLK genes (*RLK5*, *LIK1*) and one RLP gene (*AtRLP33*) were induced by SA and repressed by JA, and the known JA response disease-resistant biomarker genes *VSP1*/*2*, the JA biosynthesis gene *AOC1*, and JAZ repressor proteins *JAZ9* were induced by JA and repressed by SA. The expression of *NPR4* was consistent with the previous report that *NPR4* mRNA levels decreased rapidly following MeJA treatment, and increased following SA treatment in leaves of wild-type plants (Liu et al., 2005). *NPR4* might be involved in the cross-talk between the JA- and SA-dependent signaling pathways (Liu et al., 2005). *RPP4* required multiple immune components, including *PAD4*, *SID2* and SA to resist against *Peronospora parasitica* (van der Biezen et al., 2002). *LIK1* was phosphorylated by the chitin receptor *CERK1* and regulated PTI. The *lik1* mutants reduced JA and ethylene response genes, increased susceptible to the necrotrophic pathogen and showed resistance to the hemibiotrophic pathogen (Le et al., 2014). JAZ repressed JA response genes by binding to the MYC TFs, and this repression was relieved upon wounding or pathogen attack (Huot et al., 2014). *JAZ9* transcript levels raised during JA signaling (Thines et al., 2007). The remaining genes seemed to associate with non-immune plant processes such as ABA regulation, brassinosteroid signaling pathway, cell cycle, and ion transport and included 22 genes involved in unknown biological processes (Supplementary Table S7).

### Comparison of Differential Gene Expression Patterns Between Bio/Necrotrophic Pathogens Infection and JA/SA Analogs Treatment

In order to observe the changes of JA/SA response genes after pathogen attack, the differential expression profiles of *Arabidopsis thaliana* attacked by *Pto* DC3000, Turnip crinkle virus (TCV), *B. cinerea*, *P. rapae, S. sclerotiorum*, *aphid*, *A. brassicicola* were exhibited in Figure 3 and Supplementary Figure S2. Strikingly, the JA/SA co-induced genes were all activated by various lifestyle pathogens. Therefore, these genes might play important roles in resistance against these pathogens. Most of the JA/SA co-repressed genes were also inhibited by these biotic stressors. This suggested that growth-defense trade-off was ubiquitous in the interactions of various plant elicitors and pathogens with plants.

Virulent pathogens could hijack plant defense signaling to facilitate their infection (Zhang L. et al., 2017). There were many genes activated after necrotrophic pathogens *B. cinerea* inoculation infection, the attack of *P. rapae* and SA analogs treatment, but inhibited by JA analogs (Figure 3 and Supplementary Figure S2). Many genes were activated after hemibiotrophic pathogens *Pto* DC3000 infection and JA analogs treatment, but inhibited by SA analogs (Figure 3 and Supplementary Figure S2). These genes might be manipulated by pathogens, therefore, helped them in colonization of the host (La Camera et al., 2011). To identify these genes, Meta-Analysis was performed with the datasets of *P. rapae*, *B. cinerea*, *Pto* DC3000 versus mock. Then the results were compared with those of JA/SA analogs. 146 genes, including 2 known SA biosynthetic genes (*CBP60G*, *SARD1*), 7 R genes, 7 RLK genes, and 39 unknown function genes, were found to be upregulated by *P. rapae*, *B. cinerea*, and SA analogs, but repressed by JA analogs (Supplementary Table S8). The ontologies associated with SA signaling regulation, plant cell death (PCD) and innate immune response were overrepresented in these genes. Indeed, *B. cinerea* could produce β-(1,3)(1,6)-D-glucan, which stimulated SA accumulation and suppressed JA-mediated defenses, thereby promoting plant susceptibility to *B. cinerea* (Oirdi et al., 2011). However, SA-mediated innate immune also played important roles in plant resistance to *B. cinerea* (Zhang W. et al., 2017). Without further experimentation, it was not possible to determine whether these genes manipulated by necrotroph pathogens. On the other hand, 51 genes, including the known JA signaling genes (*AOC1*, *DDE2/AOS*, *JAZ9*, *CORI3*, *VSP1*, *VSP2*, *IGMT5*), and 9 unknown function genes, were found to be upregulated by *Pto* DC3000, and JA analogs, but repressed by SA analogs (Supplementary Table S8). The ontologies associated with JA signaling pathway, glucosinolate, and response to wounding were overrepresented in these genes. These genes might be manipulated by *Pto* DC3000 to activate JA signaling and dampen SA signaling. *Pto* DC3000 was highly virulent on *Arabidopsis thaliana* by delivering small molecules such as the JA-Ile mimic phytotoxin COR and effectors through the type III secretion system to enhance JA signaling and suppress SA-mediate immune (Xin and He, 2013). The *dde2/ein2/pad4/sid2*-quadruple mutant (*deps*) was simultaneously deficient in JA biosynthesis, ethylene, *PAD4*, and SA signaling (Mine et al., 2018). Here, almost all JA-responsive genes in *deps* were upregulated upon *Pto* DC3000 infection (Figure 3). These could attribute to COR or effectors produced by this bacterial pathogen.

Unlike *B. cinerea*, *S. sclerotiorum*, a devastating necrotrophic pathogen and causal agent of the white mold and stem rot diseases, inhibited a large number of JA/SA response genes (Figure 3 and Supplementary Figure S2). There were a few genes reported to resist against *S. sclerotiorum* (Mbengue et al., 2016). Here, the genes activated by *S. sclerotiorum* could be the candidate genes for resistance against this pathogen. There were lots of JA/SA response genes activated by TCV. These suggested that both JA/SA signals might play important roles in plant anti-viral defense, and the JA/SA analogs might be used for crop protection against plant viruses. Actually, the sequential application of JA and SA inhibits the replication of *Cucumber mosaic virus*, *Tobacco mosaic virus*, and TCV in *Arabidopsis thaliana*, tobacco, tomato and hot pepper (Shang et al., 2011).

## Discussion

JA and SA were important phytohormones that regulate a plethora of processes including plant growth and development, as well as defense against biotic invaders. However, the systematic exploration of the shared and unique biological process between SA and JA signaling was lacking. Here, 40% of all differential expression genes were regulated by JA/SA in the same or opposite direction (Figure 2). 363 JA/SA co-induced genes were also activated by biotrophic and necrotrophic pathogens (*Pto* DC3000, TCV, *B. cinerea*, *S. sclerotiorum*, *P. rapae*, and *aphid*), which were confirmed by microarray datasets, and were defined as broad-spectrum disease resistance genes (Figure 3 and Supplementary Figure S2). Most of JA/SA co-repressed genes were also inhibited by biotrophic and necrotrophic pathogens (Figures 2, 3 and Supplementary Figure S2), and involved in nitrogen storage, photosynthesis, auxin transport and gibberellin biosynthetic. This suggested that plants under treatment of plant elicitors or pathogens attack prioritized appropriate defense response over growth by activating the shared SA and JA response.

### The Possibility of Simultaneously Activating JA/SA Signals

In the challenging environment, plants encountered numerous biotrophic and necrotrophic pathogens, which required SA or JA signal to confer resistance, respectively. SA-dependent systemic acquired resistance (SAR) pathway and JA-dependent induced systemic resistance (ISR) pathway were resistant against a broad spectrum of pathogens. Simultaneous activation of SAR and ISR enhanced defense against *Pto* DC3000 (van Wees et al., 2000). JA/SA signals could also be simultaneously activated in other situations, for example, treatment with iturin A, an antifungal cyclic lipopeptide produced by *Bacillus* species, or the combination of JA and SA at low concentration (Mur et al., 2006; Kawagoe et al., 2015). These observations indicated that JA/SA pathway were compatible. Here, 60% of differential expression genes were uniquely regulated by JA or SA (Figure 2), and showed significantly distinct expression patterns spanning mock-JA or mock-SA conditions (Figures 3, 5 and Supplementary Figure S2). These not only confirmed a notation that a single group of genes would not keep co-expression spanning all different conditions, and would be modulated by different regulatory mechanisms (Polanski et al., 2014). These also showed that plants were highly plastic in their capacity to swiftly rewire their transcriptome to JA or SA treatment. Additionally, JA response marker gene *PDF1.2* and SA response marker gene *PR1* were uniquely upregulated by JA and SA, respectively, in Meta-Analysis. The promoter of *PDF1.2* responded to MeJA but not to SA (Manners et al., 1998). *TGA3*, the direct regulators of *PR1*, was present in M183 (Figure 5), where the genes showed highly coordinated co-expression across the mock-SA conditions. Thus, some downstream genes of SA signaling might be free from the impact of JA signaling activation, and so do with the downstream genes of JA signaling. So, plants could simultaneously activate JA/SA signals in some cases.

### Necrotrophs Might Suppress Defense Responses Through JA Suppressed Genes

The JA-mediated defense was vital for plants resistant against *B. cinerea*. The exogenous application of JA analogs enhances resistance of several plant species against *B. cinerea* (Zhu and Tian, 2012; Scalschi et al., 2015). Jasmonate-deficient plants showed increased susceptibility to *B. cinerea* (Rowe et al., 2010; Scalschi et al., 2015). Besides, MeJA impaired the plasma membrane integrity of *B. cinerea* spores and exhibited direct antimicrobial activity in *vitro* (Zhu and Tian, 2012). During *B. cinerea* infection of *Arabidopsis thaliana*, lots of genes involved in JA signaling were activated (Windram et al., 2012).

However, *B. cinerea* might suppress plant defense responses through JA suppression genes. For instance, *ATGRXS13* was activated after *B. cinerea* infection and SA analogs, but repressed by JA (Supplementary Tables S3, S7; La Camera et al., 2011). MeJA pretreatment reduced the induction of *ATGRXS13* by SA. Plants impaired in *ATGRXS13* showed resistance to *B. cinerea* (La Camera et al., 2011). *PSKR1*, a tyrosine-sulfated peptide receptor (Mosher et al., 2013), was also upregulated by *P. rapae*, *B. cinerea*, and SA analogs, but repressed by JA analogs (Supplementary Table S3). *PSKR1*-overexpressing plants increased resistance to necrotrophic pathogens *A. brassicicola* and enhance susceptibility to *Pto* DC3000. *PSKR1* mutants were more susceptible to the *A. brassicicola* (Mosher et al., 2013). So, it might be a virulent strategy for necrotrophs to facilitate their infection of the plant via JA suppression genes.

### Identified JA/SA Markers Are Helpful to Discover Potential Plant Elicitor

Plant elicitors were attractive and promising alternatives of conventional pesticides because they enhanced plant endogenous immunity without direct toxicity to the pathogens (Zhou and Wang, 2018). The screening strategies of synthetic elicitors could be divided into phenotypic drug discovery (PDD) approaches and pathogen-responsive-gene-based (PRGB) strategies (Bektas and Eulgem, 2015). PDD had been successfully used to discover plant elicitors, such as INA, Tiadinil, N-cyanomethyl-2-chloroisonicotinamide, 3-chloro-1-methyl-1H-pyrazole-5-carboxylic acid, Imprimatins, and sulfanilamide compounds (Bektas and Eulgem, 2015). Most of PDD approaches are involved in experimental assessment of hundreds of potential elicitor to protect against plant diseases, which was laborious, time-consuming, and costly. The SA analogs DCA was identified based on pathogen-responsive *CaBP22::GUS* reporter gene in *Arabidopsis thaliana* (Bektas and Eulgem, 2015), which was a PRGB strategy. However, most of the known synthetic elicitor belong to SA function analogs (Bektas and Eulgem, 2015). This was attributed to the compound screening strategies, mainly based SA-triggered defense responses as the indicator of defense activation (Bektas and Eulgem, 2015). Here, JA/SA function analogs were discriminated as two classes based on differential expression matrix of identified markers. OPDA and DCA, which were served as validation datasets and had a little different mode of action from JA and SA, respectively, were clustered with JA and SA analogs, respectively (Figure 6 and Supplementary Figure S3B), and showed consistent differential expression patterns with JA and SA analogs, respectively (Supplementary Figure S2). Hence, the identified genes could be used as PRGB strategies for screening potential JA/SA plant elicitors and predict the mode of action of new synthetic elicitors based on differential expression matrix.

Notably, our method may be more suitable for finding functional analogs of SA and JA, which do not entirely mimic SA or JA and have some unique signaling events. For instance, MeJA, JA-Ile and COR are JA functional analogs. JA-Ile is a major bioactive JA derivative that binds to COI1 to activate JA signaling and defense response. JA-Ile does not cause seedling growth inhibition (Zhang et al., 2019). Exogenous MeJA activates defensive systems in receiver plants by essentially converting itself into JA and JA-IIe and initiating signal transduction, leading to volatile organic compound emissions and induction of endogenous JA-IIe (Tamogami et al., 2008). MeJA suppresses plant growth (Zhang et al., 2019). COR, a toxin produced by the bacterium *P. syringae*, acts directly as an agonist of the COI1-JAZ coreceptor and induces JA-mediated defense response (Attaran et al., 2014). COR arrests plant seedling growth (Attaran et al., 2014). Although these three compounds can affect plant growth to varying degrees, they all activate the expression of JA-responsive genes and JA-mediated plant defense responses. The mainly common features of these mimics of SA or JA remain elusive. Here, we exhibited that these SA and JA analogs can be clustered the responses into two “clusters” when using PCA, and HCA based on the global differential expression matrix rather than the expression matrix. And the genes (11539 genes, one-third of *Arabidopsis thaliana* genome) identified by Meta-Analysis show consistent differential expression patterns when SA or JA analogs treatment, correspondingly (Figure 3 and Supplementary Figure S2). Taken together, although a group of mimics has their unique signaling events, the major changes of gene expression they induced have a similar tendency.

## Data Availability Statement

Publicly available datasets were analyzed in this study. This data can be found here: PRJNA224133, SRP041507, PRJNA270886, PRJNA354369, PRJNA303108, PRJNA394842, PRJNA318266, PRJNA348676, PRJNA354373, PRJNA276445, PRJNA315516, PRJNA418121, PRJNA336058, GSE39384, GSE10732, GSE51626, GSE22942, GSE10646, GSE13833, E-MEXP-3122, GSE16497, GSE5684, GSE50526, GSE17500, and GSE5520.

## Author Contributions

ZF and NZ conceived the original research plan. NZ analyzed the data, performed the statistical analyses, data visualization, and wrote the manuscript. DY and SZ helped to revise the manuscript. All authors contributed to the article and approved the submitted version.

## Conflict of Interest

The authors declare that the research was conducted in the absence of any commercial or financial relationships that could be construed as a potential conflict of interest.

## Dedication

This article is dedicated to the 101st anniversary of Nankai University.

## Supplementary Material

FIGURE S1 The batch effects from separate laboratories compromised the biological interpretation of the data. PCA plots showed the first 2 principal component diagram before the removal of batch effects. Symbol sizes increased with time (hours) after the application of JA/SA analogs. The Numbers 1–5, MeJA1-3, and COR/2 represented datasets from different laboratories. 1, SRP041507 (COR); 2, PRJNA224133 (SA/MeJA1); 3, PRJNA354369 (BTH/MeJA2); 4, PRJNA318266 (MeJA3); 5, PRJNA270886 (COR2); 6, PRJNA303108 (DPMP); 7, PRJNA394842 (INA).

FIGURE S2 Verification of the results of the Meta-Analysis with microarray datasets. Heatmap depicted the differential gene expression profiles, challenged by JA/SA analogs, biotrophic and necrotrophic pathogens. The datasets of JA/SA analogs were same as the datasets shown in Figure 2. *S. sclerotiorum* (E-MEXP-3122); *aphid* (GSE17500); *A. brassicicola* (GSE50526); *B. cinerea* (GSE5684); *Pto* DC3000^1^ (GSE5520); *Pto* DC3000^2^ (GSE17500).

FIGURE S3 The classification effect of identified SA and JA biomarker genes. **(A,B)** HCA dendrogram profiled classification of biomarker genes’ differential expression (**A**, RNA-Seq; **B**, microarray). SA1/2 represented datasets from different laboratories. SA1 (GSE22942); SA2 (GSE51626); BTH (GSE10646); INA (GSE13833); DCA (GSE13833); MeJA (GSE39384); OPDA (GSE10732); The RNA-Seq datasets are same to Supplementary Figure S1.

TABLE S1 *Arabidopsis thaliana* gene expression datasets used in this study.

TABLE S2 Differential expression genes list in SA and JA treatment identified by Meta-Analysis.

TABLE S3 The differential expression matrix of all RNA-Seq datasets in this study.

TABLE S4 GO term enrichment for the results of Meta-Analysis and selected Wigwam modules. The GO enrichment analysis was performed on the results of Meta-Analysis and the intersection genes between JA/SA regulated genes and the selected Wigwam modules.

TABLE S5 Wigwams module membership. Gene memberships of 312 Wigwams modules found by jointly mining Mock, SA, and MeJA time courses.

TABLE S6 Venn diagram information for Figure 4. The JA/SA regulated genes of the Meta-Analysis were compared with Wigwams genes using Fisher exact test.

TABLE S7 JA/SA antagonistic biomarkers identified by feature selection algorithms.

TABLE S8 The genes exhibited crossed differential expression patterns toward JA/SA analogs and bio/necrotrophic pathogens. The genes were repressed by SA but induced by JA and biotrophic pathogens, or repressed by JA but induced by SA and necrotrophic pathogens.

## Abbreviations

BTH: benzo-(1,2,3)-thiadiazole-7-carbothioic acid S-methyl ester
COR: coronatine
DCA: 3,5-Dichloroanthranilic acid
*deps*: *dde2/ein2/pad4/sid2*-quadruple mutant
DPMP, 2: 4-dichloro-6-{(E)-[(3-methoxyphenyl)imino]methyl}phenol
ETI: effector-triggered immunity
GO: Gene Ontology
HCA: hierarchical clustering analysis
INA: 2,6-dichloro-isonicotinic acid
ISR: induced systemic resistance
JA: jasmonic acid
OPDA: 12-oxo-phytodienoic acid
*P. rapae*: *Pieris rapae*
PAMPs: pathogen-associated-molecular-patterns
PCA: principal component analysis
PCD: plant cell death
PDD: phenotypic drug discovery
PRGB: pathogen-responsive-gene-based
PTI: PAMP-triggered immunity
PTI: PAMP-triggered immunity
*Pto* DC3000: *P. syringae pv. tomato* DC3000
R: resistance genes
RF: random forest
RFE-SVM: recursive feature elimination SVM
R-SVM: recursive-SVM
RuBisCO: Ribulose − 1,5 − bisphosphate carboxylase/oxygenase
SA: salicylic acid
SRA: systemic acquired resistance
SVM: support vector machines
TCV: Turnip crinkle virus
TFs: transcription factors.
