# Corrigendum: Revealing Shared and Distinct Genes Responding to JA and SA Signaling in Arabidopsis by Meta-Analysis

**DOI:** 10.3389/fpls.2020.596349

**Published:** 2020-09-17

**Authors:** Nailou Zhang, Shuang Zhou, Dongyan Yang, Zhijin Fan

**Affiliations:** State Key Laboratory of Elemento-Organic Chemistry, College of Chemistry, Nankai University, Tianjin, China

**Keywords:** plant Immunity, salicylic acid, jasmonic acid, meta-analysis, co-expression network, systems biology, molecular markers

In the original article, the supplementary material were misnamed and **Table S3** is missing. The corrected supplementary material have now been published appears below. The authors apologize for this error and state that this does not change the scientific conclusions of the article in any way. The original article has been updated.

