# Supplementary figures and images for: Revealing Shared and Distinct Genes Responding to JA and SA Signaling in Arabidopsis by Meta-Analysis
Source: Front Plant Sci. 2020 Jun 26;11:908. doi: 10.3389/fpls.2020.00908 (PMC7333171; doi:10.3389/fpls.2020.00908)

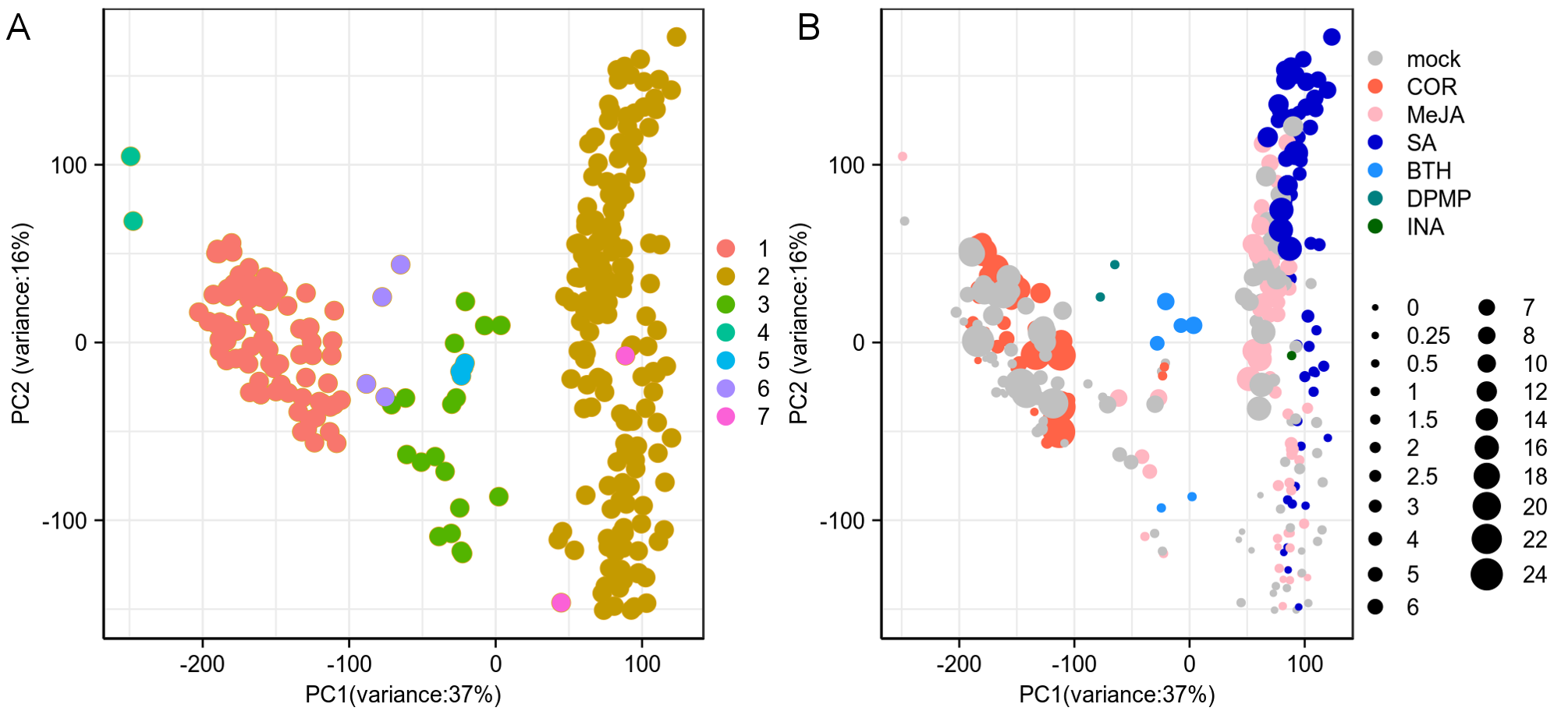

Supplement: FIGURE S1 — The batch effects from separate laboratories compromised the biological interpretation of the data. PCA plots showed the first 2 principal component diagram before the removal of batch effects. Symbol sizes increased with time (hours) after the application of JA/SA analogs. The Numbers 1–5, MeJA1-3, and COR/2 represented datasets from different laboratories. 1, SRP041507 (COR); 2, PRJNA224133 (SA/MeJA1); 3, PRJNA354369 (BTH/MeJA2); 4, PRJNA318266 (MeJA3); 5, PRJNA270886 (COR2); 6, PRJNA303108 (DPMP); 7, PRJNA394842 (INA). [file Image_1.tif]

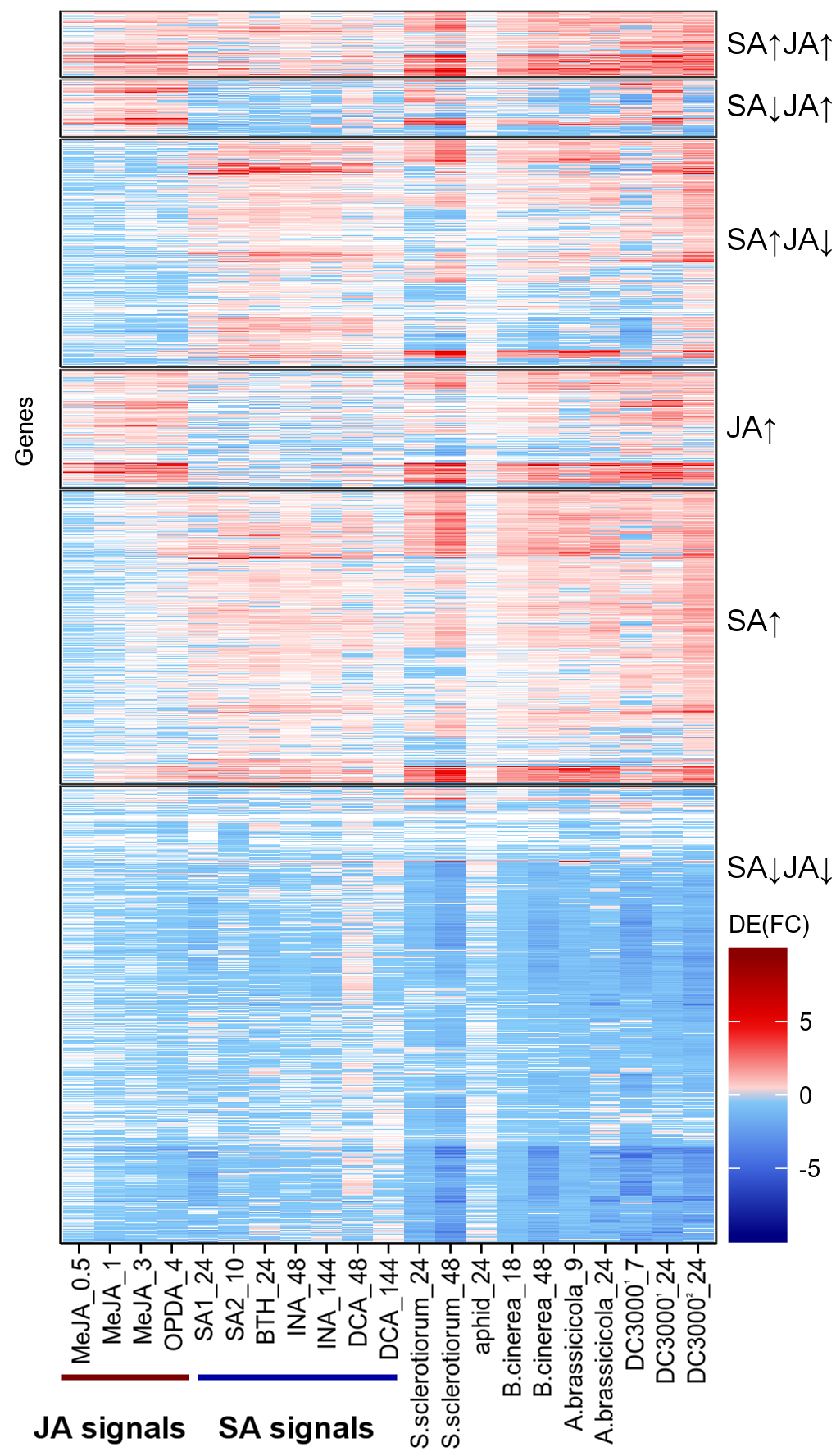

Supplement: FIGURE S2 — Verification of the results of the Meta-Analysis with microarray datasets. Heatmap depicted the differential gene expression profiles, challenged by JA/SA analogs, biotrophic and necrotrophic pathogens. The datasets of JA/SA analogs were same as the datasets shown in Figure 2. S. sclerotiorum (E-MEXP-3122); aphid (GSE17500); A. brassicicola (GSE50526); B. cinerea (GSE5684); Pto DC30001 (GSE5520); Pto DC30002 (GSE17500). [file Image_2.tif]

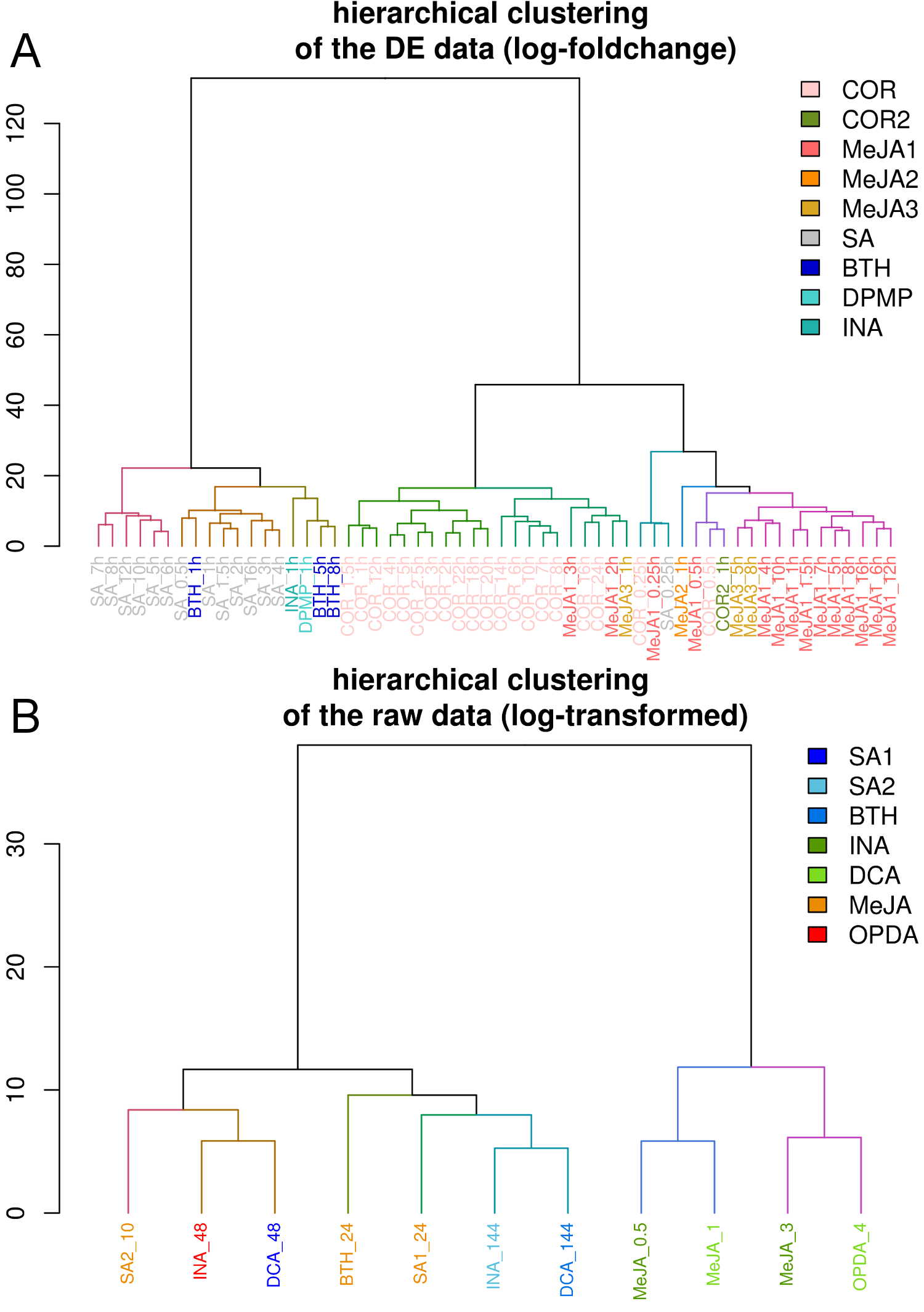

Supplement: FIGURE S3 — The classification effect of identified SA and JA biomarker genes. (A,B) HCA dendrogram profiled classification of biomarker genes’ differential expression (A, RNA-Seq; B, microarray). SA1/2 represented datasets from different laboratories. SA1 (GSE22942); SA2 (GSE51626); BTH (GSE10646); INA (GSE13833); DCA (GSE13833); MeJA (GSE39384); OPDA (GSE10732); The RNA-Seq datasets are same to Supplementary Figure S1. [file Image_3.tif]
