# Supplementary material for: Revealing Shared and Distinct Genes Responding to JA and SA Signaling in Arabidopsis by Meta-Analysis
Source: Front Plant Sci. 2020 Jun 26;11:908. doi: 10.3389/fpls.2020.00908 (PMC7333171; doi:10.3389/fpls.2020.00908)
Supplement: TABLE S1 — Arabidopsis thaliana gene expression datasets used in this study. [file Table_1.docx]

Table S1. *Arabidopsi*s gene expression data sets used in this study.

| Data Set | Treatment & genetype | Time (h) | Biological Repeat | Platforms | Meat-  analysis | Feature selection | Validation |
| --- | --- | --- | --- | --- | --- | --- | --- |
| PRJNA224133 | SA, MeJA,  mock | 0, 0.25, 0.5, 1, 1.5, 2, 3, 4, 5, 6, 7, 8, 10, 12, 16 | 4 | Illumina HiSeq 2000 | **√** | **√** |  |
| SRP041507 | COR, mock | 0, 0.25, 0.5, 1, 1.5, 2, 2.5, 3, 4, 5, 6, 7, 8, 10, 12, 14, 16, 18, 20, 22, 24 | 2 | Illumina HiSeq 2000 | √ | √ |  |
| PRJNA270886 | COR, mock | 1 | 2 | Illumina HiSeq 2500 | √ | √ |  |
| PRJNA354369 | BTH, MeJA, mock | 1, 5, 8 | 2 | Illumina HiSeq 2500 | √ | √ |  |
| PRJNA303108 | DPMP, mock | 1 | 2 | Illumina HiSeq 2500 | √ | √ |  |
| PRJNA394842 | INA, mock | 1 | 1 | Illumina HiSeq 2000 |  | √ | √ |
| PRJNA318266 | MeJA, mock | 1 | 1 | Illumina HiSeq 2000 |  | √ | √ |
| PRJNA348676 | wild type + *Pto* DC3000, deps + *Pto* DC3000, mock | 4, 6, 9, 12, 16, 24 | 3 | Illumina HiSeq 2500 | √ |  | √ |
| PRJNA354373 | *Pto* DC3000, mock | 24 | 3 | Illumina HiSeq 2500 | √ |  | √ |
| PRJNA276445 | *B. cinerea*, mock | 14 | 2 | Illumina HiSeq 2500 | √ |  | √ |
| PRJNA315516 | *B. cinerea*, *P.rapae, mock* | 6, 12, 18, 24 | 3 | Illumina HiSeq 2000 | √ |  | √ |
| PRJNA418121 | *S. Sclerotiorum,* mock | 24 | 3 | Illumina HiSeq 2500 |  |  | √ |
| PRJNA336058 | TCV, mock | 168 | 3 | Illumina HiSeq 2000 |  |  | √ |
| GSE39384 | MeJA, mock | 0.5, 1, 3 | 2 | Affymetrix |  |  | √ |
| GSE10732 | OPDA, mock | 4 | 3 | Affymetrix |  |  | √ |
| GSE51626 | SA, mock | 24 | 2 | Affymetrix |  |  | √ |
| GSE22942 | SA, mock | 10 | 3 | Affymetrix |  |  | √ |
| GSE10646 | BTH, mock | 24 | 3 | Affymetrix |  |  | √ |
| GSE13833 | DCA, INA, mock | 48, 144 | 3 | Affymetrix |  |  | √ |
| E-MEXP-3122 | *S. Sclerotiorum*, mock | 24, 48 | 3 | Affymetrix |  |  | √ |
| GSE16497 | Aphid, mock | 24 | 3 | Affymetrix |  |  | √ |
| GSE5684 | *B. cinerea,* mock | 18, 48 | 2 | Affymetrix |  |  | √ |
| GSE50526 | *Alternaria brassicicola*, mock | 9, 24 | 3 | Affymetrix |  |  | √ |
| GSE17500 | *Pto* DC3000, mock | 24 | 3 | Affymetrix |  |  | √ |
| GSE5520 | *Pto* DC3000, mock | 7, 24 | 3 | Affymetrix |  |  | √ |

(I) we confirmed the robustness of our methods by reproducing the results originating from the RNA-Seq Data by using independent Microarray Data with the same data process; (II) we exhibited that the overall differential expression pattern of RNA-Seq of INA and MeJA (the Data not used for Meta-Analysis) were consistent with the results of Meta-Analysis, which confirmed the robustness of MetaDE;
